# Metasurface-based broadband hologram with high tolerance to fabrication errors

**DOI:** 10.1038/srep19856

**Published:** 2016-01-28

**Authors:** Xiaohu Zhang, Jinjin Jin, Yanqin Wang, Mingbo Pu, Xiong Li, Zeyu Zhao, Ping Gao, Changtao Wang, Xiangang Luo

**Affiliations:** 1 State Key Laboratory of Optical Technologies on Nano-Fabrication and Micro-Engineering, Institute of Optics and Electronics, Chinese Academy of Science, P.O. Box 350, Chengdu 610209, China; 2 University of Chinese Academy of Sciences, Beijing 100049, China

## Abstract

With new degrees of freedom to achieve full control of the optical wavefront, metasurfaces could overcome the fabrication embarrassment faced by the metamaterials. In this paper, a broadband hologram using metasurface consisting of elongated nanoapertures array with different orientations has been experimentally demonstrated. Owing to broadband characteristic of the polarization-dependent scattering, the performance is verified at working wavelength ranging from 405 nm to 914 nm. Furthermore, the tolerance to the fabrication errors, which include the length and width of the elongated aperture, the shape deformation and the phase noise, has been theoretically investigated to be as large as 10% relative to the original hologram. We believe the method proposed here is promising in emerging applications such as holographic display, optical information processing and lithography technology etc.

Since it was firstly proposed to utilize the interference between the scattered light of an object and the reference beam to record the information of three-dimensional (3D) objects, holography has enjoyed continuous scientific interest. Recording and recurring the phase and amplitude of light fields are two crucial steps for the holography. In traditional holography, recording includes exposal, developing, fixation and the hologram should be illuminated with a correlative light in recurring process, then the image of the object could be observed at some space range. Traditional holography has been researched for several decades and a great deal of productions could be found in the market, but the applications of the holography are still limited by the componential materials, and the working wavelength range is rather narrow^1^,^2^. As a result, new materials with broader working wavelength range would have huge potential applications in the future holography technologies.

Recently, metamaterials have been proposed as natural candidates to enable the realization of many novel phenomena and functionalities, owing to their large flexibility to engineer the electromagnetic properties. The past decade has seen a great deal of prominent examples, such as negative refraction^3^, selective absorption and emission^4^,^5^, zero refractive index^6^, Fano resonance^7^,^8^, invisibility cloaks^9^,^10^, super-resolution imaging^11^,^12^, spinning light^13^,^14^ and phase holography^15^. Despite the success of metamaterials in a series of novel physical phenomena, using of them for practical applications is still a challenge^16^,^17^. Elaborate fabricating technology and precise alignment between different layers seem to be the main obstacles in the visible regime. Fortunately, the two-dimensional (2D) metamaterials, i.e., metasurfaces have been demonstrated to be able to control light in a flat surface. Consisted of different patterned elements, metasurfaces possess great flexibility to control the phase and amplitude of the electromagnetic wave via a single layer^18^. Despite it is a new member in the “meta” family, an enormous interest has been attracted due to the promise for the thinner electromagnetic devices, for examples, photonic spin Hall effect generator^19^, various meta-lenses^20^,^21^,^22^, flat hologram^1^,^2^,^23^,^24^,^25^, helicity-dependent surface plasmon excitation^26^, broadband virtual shaping^27^, multicolor hologram^28^, multiple wavefront shaping^29^ and vertical split-ring resonator^30^. Nevertheless, the tolerance to the fabrication has yet to be fully investigated.

In this paper, a metasurface configuration of 2D holography which can bear high fabrication tolerances in a broad wavelength region ranging from 405 nm to 914 nm was experimentally demonstrated. By changing the orientation of each nanostructure, the phase shift can be modulated from 0 to 2π. Simultaneously, the amplitude keeps almost the same. Benefitting from the stability of the design algorithm and the robust phase change of the structure, the fabrication tolerance could be as high as 10% relative to the original designed phase hologram. A lot of structures, such as metallic nanorods^23^, elliptical nano-aperture^31^ and its Babinet-inverted structures, can hold this characteristic. The elongated aperture, which can be processed conveniently by the focused ion beam (FIB), is used to demonstrate the broadband and high tolerance feature. This design holds promise for practical systems of holographic display as the working wavelength range of the configuration covers the whole visible light region.

## Results

### Optical properties of the elongated nanoantenna

**Figure 1:**
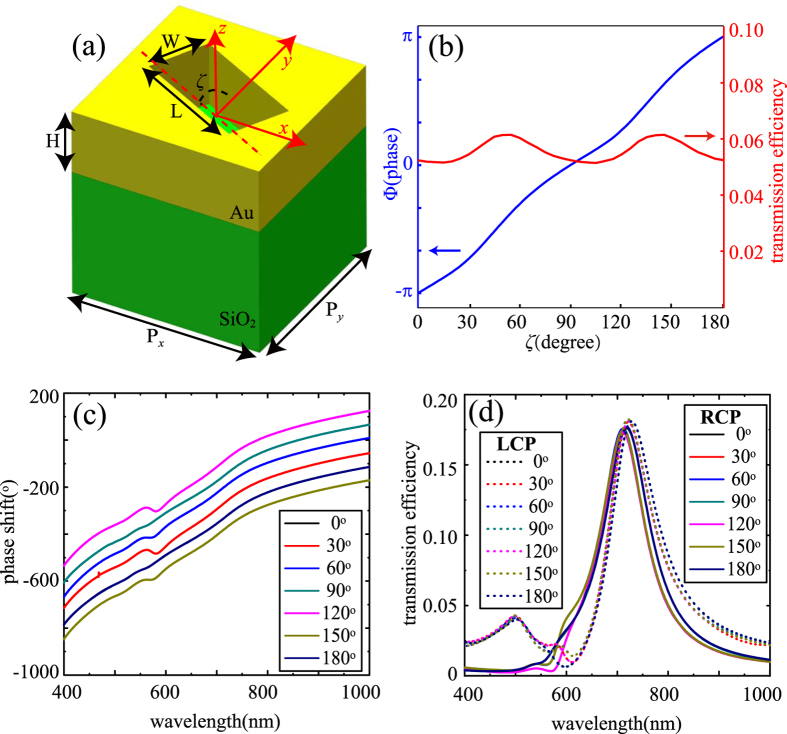
Optical performance of the elongated nanoaperture. (**a**) An illustration of the unit cell. The elongated aperture could rotate in the *x-y* plane with an orientation angle of *ζ* to produce a particular phase delay. The periods P_x_ and P_y_ are both 300 nm. The length, width and height are L = 200 nm, W = 100 nm and H = 120 nm. The thickness of the glass substrate in the experiment is 2 mm. (**b**) Phase and transmission efficiency in power versus the rotation angle *ζ* of the unit cell. The blue line and red line correspond to the phase-angle relation and transmission efficiency-angle relation, respectively. The wavelength is set with 632.8 nm. (**c**) Phase shift of the transmitted RCP light and (**d**) the transmission efficiencies of LCP and RCP light with different orientation angle *ζ* when the incident wavelength from 400 nm to 1000 nm, the colorful solid line and dot line correspond to the transmitted RCP and LCP light, respectively. The incident light is set with LCP light. The lines for 0 degree and 180 degrees are overlapped.

The schematic of unit cell is shown in Fig. 1a. The Au film perforated with elongated nanoaperture is placed on a SiO_2_ substrate. Commercial software CST MWS is applied to simulate the phase and amplitude of the transmitted light. When the unit cell is illuminated with a circular polarized plane wave with wavelength of 632.8 nm, the cross-polarized light is detected in the output field. In the following, the left-handed circular polarization (LCP) plane wave is used as input light both in the simulation and experiment. Figure 1b shows the phase and transmission efficiency in power versus the orientation angle *ζ* for the right-handed circular polarization (RCP). Clearly, the relation between Φ and *ζ* is approximately linear, agreeing well with the theoretical anticipation Φ = 2σ*ζ*, where σ = ±1 denotes the LCP and RCP. Since the phase is about twice of the orientation angle of the nano-aperture, so the phase could be modulated from 0–360 degrees. The transmission efficiency changes probably from 5.1% to 6.1% with *ζ* changing from 0 to 180 degrees. Such a little change can be neglected, which further proved that the structure could be used for the realization of phase hologram. The broadband character is shown in Fig. 1c,d. As we expected, the phase shift is mainly determined by the orientation angle at all the wavelengths. The biggest disparities of the wavelength 405 nm, 532 nm and 914 nm to 632.8 nm are about 0.12π, 0.13π and 0.05π, respectively. Although the transmission efficiencies of the RCP light change obviously with the shift of the wavelength, the transmission efficiency at a single wavelength with different orientation angle *ζ* is almost the same. This ensures a good behaviour of a phase hologram consisted of the elongated apertures. It should be noted that the LCP light without phase shift should be cleaned up in case of its influence to the image’s quality (Fig. 1d).

### Design and measurement of the metasurface hologram

**Figure 2:**
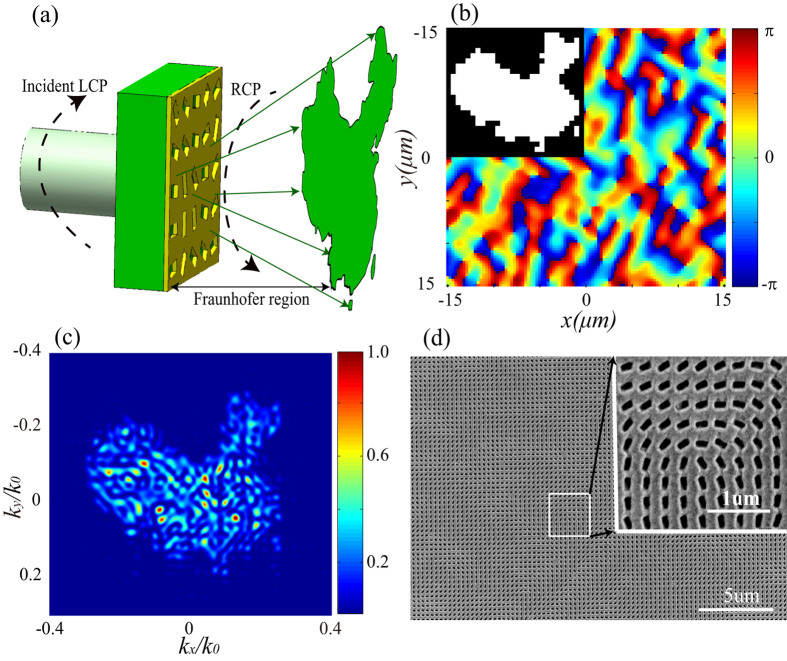
Simulation and fabrication of the sample. (**a**) Schematic diagram of the designed meta-hologram under LCP illumination and image with RCP in the Fraunhofer region. The map of China was drawn with Adobe Illustrator CS6 software (http://www.adobe.com/products/illustrator.html). (**b**) The designed phase hologram with 100 × 100 pixels for the China map shown in the inset. (**c**) The simulated holographic diffraction image acquired by CST. The intensity has been normalized. (**d**) Scanning electron microscope (SEM) image of the processed meta-hologram sample and the correlative scale bar is 5 μm. The up-right inset is a higher magnification image and the correlative scale bar is 1 μm.

Figure 2a is the operating principle of the metasurface hologram. LCP is normally incident on the metasurface from the glass substrate side. An iterative Fourier-transform algorithm (IFTA) is used to obtain the phase distribution of the hologram^32^. Figure 2b shows the designed phase hologram of a preset map depicted in the inset. The phase profile could be achieved by arranging the elongated apertures shown in Fig. 1a according to the Φ-*ζ* relation. The Finite Integration Technique (FIT) in CST MWS is used to investigate the performance of the nanoapertures array. As illustrated in Fig. 2c, we calculated the angular distribution of the hologram in the far field by using a Fourier transform of the RCP component of the electric fields given by CST. Owing to the limited size of the sample, the intensity distribution in the map’s image is not smooth enough. Nevertheless, the profile of those points is still in accord with the preset map, which could be further increased by using larger sample area. In the following, the FIB is used to fabricate the metasurface in an area of 30 μm × 30 μm (Fig. 2d).

**Figure 3:**
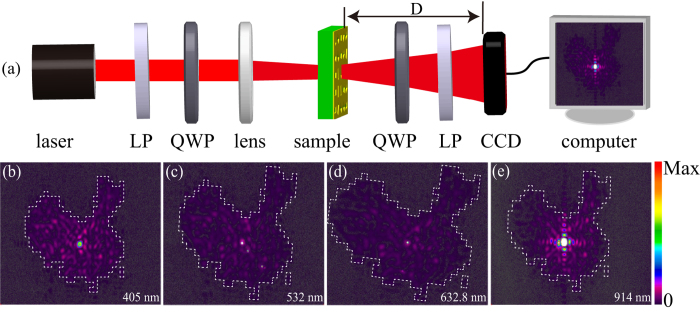
Experimental set-up and measured results. (**a**) The experimental set-up for the reconstruction of the holographic images. (**b–e**) Farfield intensity images obtained at the wavelength of 405 nm, 532 nm, 632.8 nm and 914 nm. The distances (D) between the hologram and the charge coupled device (CCD) are 6.8 cm, 6.0 cm, 6.0 cm and 3.3 cm, respectively. The white dashed lines denote the designed target maps’ boundary.

To characterize the performance of the fabricated meta-hologram, the sample was measured in an optical setup as shown in Fig. 3a. Several light sources, with wavelength of 405 nm, 532 nm, 632.8 nm and 914 nm are used to test the broadband function of the metasurface. A linear polarizer (LP) and a quarter wave plate (QWP) are placed in front of the sample to generate the desired LCP incidence. To increase the light power incident on the metasurface, a lens with long focal length is placed before the sample. As can be seen in the Fig. 1d, the efficiencies of the directly transmitted LCP light without phase shift are comparable with the efficiencies of RCP light. So another pair of QWP and LP is positioned after the hologram in order to filter the LCP background light and make the RCP light image on the charge coupled device (CCD).

Figure 3b–e shows the experimental images captured by CCD for the transmitted RCP light at wavelengths of 405 nm, 532 nm, 632.8 nm and 914 nm, respectively. Although the metasurface structure exhibits a nearly dispersionless phase distribution of transmitted cross-polarization light, the dimension of angular spectrum distribution would be affected by the shift of the wavelength. Furthermore, due to the CCD’s limited effective photosensitive area, the distance between the sample and the CCD would change with the shift of the wavelength so that the CCD could capture the whole image. That is the reason why the distances of 6.8 cm, 6.0 cm, 6.0 cm and 3.3 cm are used for 405 nm, 532 nm, 632.8 nm and 914 nm, respectively. The angular spectrum distribution of 632.8 nm is more than 0.5 *k*_0_ × 0.45 *k*_0_ as shown in Fig. 2c, so the size of the image is larger than 3 cm × 2.7 cm, which could be observed easily even with naked eyes.

Obviously, the intensity distributions of the experiments are well matched with the simulation in Fig. 2c. The measured transmission efficiencies at the wavelengths of 532nm and 632.8nm are 0.8% and 1.8%, respectively, which matches with the theoretical efficiencies shown in Fig. 1d. The sizes of the images, corresponding to 405 nm, 532 nm and 632.8 nm, are gradually enlarged as the increasing of the wavelength. Those results are in accord with the diffraction theory. Theoretically, with different incident wavelengths, the locations of the holographic images could be depicted as^23^:


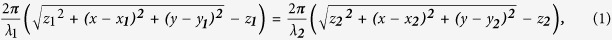


where (*x, y*) represent the coordinates of the meta-hologram and (*x*_i_, *y*_i_, *z*_i_) are the coordinates correspond to the image of the hologram at wavelength λ_i_. Considering 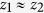 and using paraxial approximation, equation (1) could be simplified as 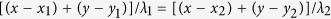. Thus, the size of the image is approximately proportional to the wavelength. This simple formula explains the experiments shown in Fig. 3b–d. Note that there is not enough space for the QWP and LP pair to eliminate the incident LCP for the wavelength of 914 nm, so a cross bright spot occurred in the center of the holographic image. Nevertheless, the images in Fig. 3b–d also have small bright spots in the centers, which may be caused by the incident LCP light is not pure enough and the sample has some fabrication errors.

## Discussion

It is important to keep a large fabrication tolerance to ensure the performance of the fabricated sample. Our metasurface hologram has very large tolerance to the fabrication as a result of the following two factors: the stability of the holographic design algorithm and the robust phase modulation character.

**Figure 4:**
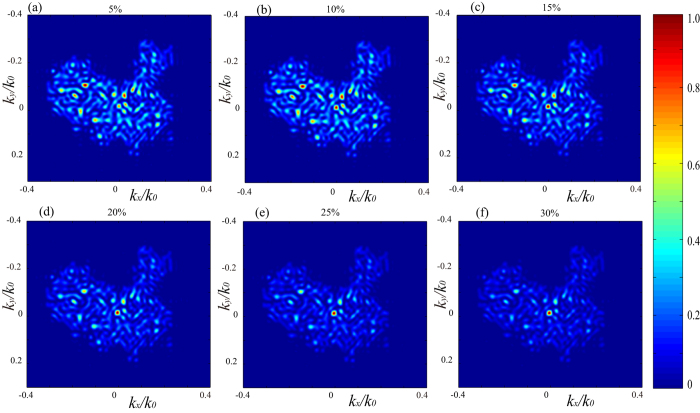
Simulation of the effect induced by phase noise. (**a–f**) Far-field intensity distributions with 5%, 10%, 15%, 20%, 25%, and 30% phase noise (relative to the original phase profile). All the intensity has been normalized.

Firstly, the holographic image is stable when a stochastic phase noise, whose extent is relative to the original phase hologram, is added on the phase hologram. In principle, the phase noise would be caused by the error in the nano-apertures’ orientation angles which are almost linear relation with the phase change. In the simulations, a relative noise of 5% to 30% is added to the original phase hologram calculated by IFTA. As shown in Fig. 4, the contrast between the map and the background is slowly reduced as the increase of the noise. Furthermore, the intensity of the bright spot in the center of the map is also slowly increased, owing to the interference effects of the modulated electromagnetic field. That is a reason why a bright spot has been obtained experimentally in the center of the map as shown in Fig. 3b–d. As the simulation results shown in Fig. 4, it can be concluded that the map could be detected without significant degradation of the performance, even if the noise is as large as 10% of the original hologram.

**Figure 5:**
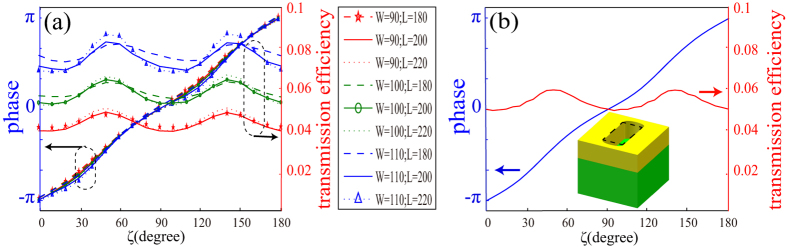
Influence of the length, width and surface shape fabrication errors. (**a**) The phase and transmission efficiencies for different wide (W) and length (L). (**b**) The phase and transmission efficiency corresponding to the changing from ideal rectangle shape to elliptic cylinders. The inset is the unit cell used in CST and the wavelength is set as 632.8 nm.

Another fabrication error is that it is a difficult task to precisely control an elongated aperture’s length and width in the fabrication process. But this fabrication error does not change the phase obviously, as shown in Fig. 5a. The phase shifts introduced by the changing of the length (L) and width (W) can be neglected. Although the transmission efficiency has a clear fluctuation, it does not seriously affect the hologram result.

Finally, it is difficult to fabricate ideal rectangle shape with FIB or electron beam lithography (EBL). Nevertheless, the effect of this fabrication distortion on the hologram can be ignored as shown in the following error analysis. All the other sizes are the same as shown in Fig. 1a except two elliptic cylinders add to the ends of elongated aperture. Figure 5b shows the phase and the transmission efficiency versus the orientation angles. Compared with Figs 1b, 5b shows that the relation of the phase and orientation angle is almost the same. The transmission of the changed structure is lower than the foregoing one, but its immanent disparity between the maximal and the minimal value of the transmission efficiency changed smaller. This result shows that changing from ideal rectangle shape to elliptic cylinders wouldn’t affect the holographic image dramatically.

According to the above analysis, one could conclude that the phase change is only the function of the orientation angle of the elongated apertures. If there are some fabrication errors in the length and width of the aperture or the processed surface shape is not ideal rectangle shape, a shift of the resonance frequency and scattering amplitude may occur, but the phase change is almost not affected. In addition, the amplitude-based holograms with carbon nanotubes^33^ use the binary amplitude array while our hologram using continuous phase profiles which can acquire a better quality image in theory and the multilayer metamaterial hologram at infrared wavelengths^15^ requires complex fabrication technology including precise alignment between different layers. Thus, compared with other 2D holographic techniques, the structure used here can realize the subwavelength pixel sizes with multi-level phase profiles more easily.

In conclusion, we have demonstrated a metasurface hologram in a broad wavelength range containing the whole visible and the near-infrared range. It is the first time to use the elongated nanoaperture, the complementary structure of nanorod antenna, to realize the hologram. The phase changes rely on the phase discontinuousness between the incident LCP light and the across polarization of RCP light. The hologram structure is capable of tolerating a large fabrication imperfection as big as 10% noise, which comprises the length and width of the rectangle aperture, the shape deformation of the rectangular aperture as well as the phase noise. Such a scheme has a huge potential application in the production of holographic display, optical information processing and optical storage with multiple wavelengths etc.

## Methods

### Numerical simulation

Iterative Fourier-transform algorithm is used to design the hologram. The hologram contains 100 × 100 pixels then its dimension is 30 μm × 30 μm. A commercial full-wave electromagnetic software (CST Microwave Studio) and Fraunhofer diffraction formula are used to verify the design.

### Sample fabrication

All the samples were fabricated on 1 mm thick quartz substrates. A 3-nm-thick Cr film and 120-nm-thick Au film were subsequently deposited on the cleaned substrates by magnetron sputtering in a same sputter chamber. The elongated apertures were then milled on the Au/Cr film using a Ga+ focused ion beam (FIB, FEI Helios Nanolab 650).

## Additional Information

**How to cite this article**: Zhang, X. *et al*. Metasurface-based broadband hologram with high tolerance to fabrication errors. *Sci. Rep.* **6**, 19856; doi: 10.1038/srep19856 (2016).
